# Targeting Regulatory T Cells to Treat Patients With Systemic Lupus Erythematosus

**DOI:** 10.3389/fimmu.2018.00786

**Published:** 2018-04-17

**Authors:** Masayuki Mizui, George C. Tsokos

**Affiliations:** ^1^Department of Nephrology, Osaka University Graduate School of Medicine, Suita, Osaka, Japan; ^2^Division of Rheumatology, Department of Medicine, Beth Israel Deaconess Medical Center and Harvard Medical School, Boston, MA, United States

**Keywords:** systemic lupus erythematosus, regulatory T cells, tissue Treg, low-dose IL-2 treatment, immunometabolism

## Abstract

Regulatory T cells (Tregs) are central in integration and maintenance of immune homeostasis. Since breakdown of self-tolerance is a major culprit in the pathogenesis of systemic lupus erythematosus (SLE), restoration of the immune tolerance through the manipulation of Tregs can be exploited to treat patients with SLE. New information has revealed that Tregs besides their role in suppressing the immune response are important in tissue protection and regeneration. Expansion of Tregs with low-dose IL-2 represents an approach to control the autoimmune response. Moreover, control of Treg metabolism can be exploited to restore or improve their function. Here, we summarize the function and diversity of Tregs and recent strategies to improve their function in patients with SLE.

## Introduction

Breakdown of self-tolerance is critical in the development of systemic lupus erythematosus (SLE) (1). Innate and adaptive immune responses against self-antigen induce the production of autoantibodies and the deposition of immune-complexes in tissues leads to the activation of complement, accumulation of neutrophils and monocytes, and self-reactive lymphocytes (2). The variety of clinical manifestations may reflect the multiple and heterogeneous pathways that account for the expression of disease (3). Chronic inflammation caused by the immune response against self-antigens leads to the development of irreversible damage in tissues including the kidney. Efforts to resolve or contain the inflammatory response include curtailing autoantibody production and the levels of type I interferon and various chemoattractants (4, 5). Belimumab, the soluble B-lymphocyte stimulator (BAFF) blocking antibody, which has been approved by FDA to treat patients with SLE has marginal clinical efficacy and only in patients with moderate disease. However, *post hoc* analysis and long-term follow-up studies have revealed that belimumab does not induce a rapid clinical benefit and the clinical efficacy seems to be limited (6). Recent advances in our understanding of regulatory T cell (Treg) physiology and metabolism have fueled new therapeutic strategies which involve the improvement of Treg function for the treatment of SLE and other autoimmune diseases as well as transplant rejection and cancer (7, 8). Low-dose IL-2 supplementation was first shown to expand Tregs and improve clinical manifestations in patients with chronic graft-versus-host disease (cGVHD) and hepatitis C virus-associated vasculitis (9, 10). Low-dose IL-2 therapy has been claimed in case reports and non-controlled studies to improve Treg numbers and clinical manifestations in patients with SLE (11, 12). Ongoing clinical trials (NCT03312335: Charact-IL-2, NCT 01988506: TRANSREG) will test the therapeutic value of low-dose IL-2 in patients with SLE. Therefore, a better understanding of the molecular events which account for the poor function of Tregs in patients with SLE along with their poor response to IL-2 is needed to optimize therapeutic approaches. Further, it should be clarified how Tregs may contribute to containing tissue inflammation or repair organ damage. Tregs can modulate the function of the immune system as well as the function of non-lymphoid organs through the acquisition of tissue-defined gene expression.

## Pleiotropic Effects of Tregs

### Treg Subsets and Tissue Tregs

Self-tolerance is accomplished with the deletion of self-reactive lymphocytes during development. However, self-reactive T cells escape negative selection in the thymus and persist in the periphery (13), where Tregs are important gatekeepers in preventing aberrant activation of self-reactive lymphocytes. Tregs develop in the thymus (tTreg) through strong T cell receptor (TCR) signaling just below the threshold for negative selection. Therefore, Tregs recognize self-antigens for their differentiation. Tregs are also induced from naïve CD4^+^ T cells (pTreg) (14). In the human, peripheral blood Tregs may be present in resting (Foxp3^+^CD45RA^+^CD25^+^CD127^−^) or activated/memory (Foxp3^++^CD45RA^−^CD25^++^CD127^−^) phenotype (Table 1) (15). A subset of memory Tregs is known as T helper-like Tregs which can be further classified, depending on the kind of environmental stimuli, as T helper-like Tregs type1 (TH1), TH2, TH17-like, and T-follicular regular populations which co-express T-bet, GATA3/IRF4, RORγt, and Bcl6, respectively (16, 17). These T helper-like memory Tregs share chemokine receptors with individual T helper cells and are thought to be distributed into the appropriate site of each class of the immune response (13). Besides the conventional Tregs, CD4^+^Foxp3^−^ type 1 T regulatory (Tr1) cells expressing IL-10 were recently identified and shown to display strong immunosuppressive activity and to be involved in the maintenance of tolerance (18–20).

**Table 1:** Genes and phenotypes of regulatory T cells detected in humans.

Tissue	Phenotype	Foxp3	CD45RA	CD25	CTLA4	GITR	ICOS	CD127	Others genes	References
Blood	Resting/naive	+	+	+	−	−	−	−		(15)
	Activated/memory	++	−	++	+	+	+	−	T-bet, GATA-3, RORγt, Bcl-6	(13, 16, 17)
	Type 1 T regulatory	−	−	−	n.d.	n.d.	+	−	IL-10, CD49b, LAG3, AhR	(18–20)
Skin	Memory	+	−	++	+	+	+	−	IL-17	(46)
Lung	Activated	+	−	+	+	−	+	−		(30)
Colon	Effector	++	−	++	+	+	n.d.	−	IL-17, RORγt, CD49d, CD103	(22, 31)
Visceral adipose tissue	Activated	+	−/+	+	n.d.	n.d.	n.d.	−	ST2, OX40	(25, 32, 34)
Joint	Activated	+	−	++	n.d.	n.d.	n.d.	−	IL-17, CD161	(33)

*n.d., not determined*.

In tissues, Tregs are more abundant, percentage-wise, than in the peripheral blood and most of tissue-resident Tregs have an activated/memory phenotype (21). Moreover, gene expression patterns in tissue-resident Tregs depend distinctly on the hosting tissue. For example, an intestinal pTreg subset expresses RORγt and can produce IL-17 (22) and visceral adipose tissue (VAT) Tregs express PPARγ to regulate insulin sensitivity (23, 24). VAT Tregs also highly express IL-33 receptor (ST2), a receptor for alarmin that induces T_H_2 responses, which is required for Treg accumulation into VAT (25). Tregs seem to be formed in the thymus by the time of birth but they diverge dependent on the tissue environment. Perinatally generated tTregs that are Aire-dependent translocate into tissues and persist to maintain self-tolerance (26). In addition, some strains of gut microbiota can induce Tregs in intestine (27). Therefore, colonic Tregs originate from both tTreg and pTreg but VAT and muscle Tregs are reported to be of thymic origin (tTreg) (28). The biology and characteristics of human tissue-resident Tregs have been reviewed (29) and are summarized in Table 1 (30–34).

### Immunosuppressive Aspects of Tregs

The suppressive action of Tregs on effector T cells (Teffs) is well established. First, Tregs inhibit Teff expansion by consuming local IL-2 because they express higher levels of CD25 (35). Second, Tregs inhibit Teffs in a contact-dependent manner. Tregs downregulate the expression of costimulatory ligands CD80/86 on antigen-presenting cells through trans-endocytosis. This is accomplished by CTLA4 which is expressed on Tregs and binds to CD80/86 with higher affinity than CD28 (36). Furthermore, Tregs can deprive energy factors from Teff cells. CTLA4-mediated signals induce indoleamine 2,3-dioxygenase in antigen-presenting cells resulting in the starvation of Teff cells (37, 38). A subset of Treg expresses an ectonucleotidase CD39 which catalyzes the degradation of proinflammatory molecule adenosine triphosphate (ATP) into adenosine diphosphate (ADP) and adenosine monophosphate (AMP) and dampens Teff activation and proliferation (39). In the resting state, Tregs localize in clusters of IL-2-producing T cells that are activated by self-antigen within secondary lymphoid tissues (35). Many other different mechanisms of suppression have been documented and are summarized in Table 2 (38, 40). Recently, Tregs were shown to suppress autophagy in antigen-presenting cells and thus limit the production of autoantigens (41).

**Table 2:** Modes of action of Tregs.

Target	Modes of actions	Reference
Effector T cells	IL-2 consumption to inhibit clonal expansion	
	Suppressive cytokine secretion (TGFβ, IL-10, IL-35)	(38)
	Hydrolysis of adenosine phosphates *via* CD39, CD73	(39)
	Direct cell killing *via* perforin/granzyme	(40)

Dendritic cells/antigen-presenting cells	Blocking CD80/86 through CTLA4	(36)
	Inhibition of autophagy through CTLA4	(41)
	Indoleamine 2,3-dioxygenase induction	(37)

Hair follicle stem cells	Promoting proliferation and differentiation	(48)
Muscle progenitor cells	Amphiregulin-mediated differentiation	(43)

Adipocytes	Maintain insulin tolerance through IL-10	(23)

### Tregs in Wound Repair and Tissue Regeneration

Neutrophils and myeloid mononuclear cells such as monocytes infiltrate injured tissues in early phases. Monocytes differentiate into M1-type macrophages which are involved in the clearance of apoptotic and necrotic cells and debris and subsequently to M2-type macrophages which become involved in matrix remodeling and promotion of angiogenesis and tissue regeneration (42). Lymphocytes, including CD4^+^ and CD8^+^ T cells, are also recruited to the sites of inflammation and have been thought to promote tissue injury. Recent reports though have demonstrated that CD4^+^Foxp3^+^ Tregs accumulate in the skeletal muscle after injury on time to switch from proinflammatory to the proregenerative (43). The Treg population persists at high numbers even 1 month after the injury. Notably, these Tregs express high levels of amphiregulin (Areg), an epithelial growth factor (EGF) family protein, and promote muscle regeneration through the EGF receptor (EGFR) signaling axis (43). Tregs expressing Areg can protect lungs from infection-induced damage (44). In addition, Tregs also express EGFR and the Areg-EGFR axis is critical for the local Treg function. Areg is produced not only by Tregs but by Th2 cells and other myeloid cells including mast cells and control the immune response by regulating Treg cell function (45). Involvement of Areg-EGFR signals in Treg-mediated tissue regeneration is also observed in skin injury by promoting wound healing (46, 47). Furthermore, the same group recently demonstrated that skin Tregs preferentially reside close to hair follicle stem cells (HFSCs) and help HFSC-mediated hair regeneration (48). More recently, Tregs were demonstrated to promote directly myelin regeneration in the central nervous system indecently of immunomodulation (49).

## IL-2, Tregs, and SLE

### IL-2 Deficiency and Impaired Treg Function in SLE

While IL-2 is critical for the differentiation and function of Tregs, it is a well-known fact that IL-2 production by conventional T cells (Tconv) is impaired in SLE (50). IL-2 gene is silenced through transcriptional regulator, cyclic AMP response element modulator alpha (CREMα), which is overexpressed by SLE Tconv cells. Repression of IL-2 also caused by enhancement of calcium/calmodulin-dependent kinase IV (CaMK4) (51) and decrease of serine/arginine-rich splicing factor 1 (52, 53). The absence of IL-2 probably favors differentiation and expansion of IFNγ-producing T_H_1 cells and IL-17-producing T_H_17 cells, accumulating in organs such as the skin and the kidney (54, 55). Regulatory T cell numbers decrease in lupus-prone mice as they age and the disease progresses (56). In humans, several studies have analyzed the frequency of Tregs in SLE and reported conflicted results (57). The reported discrepancies may be due to the applied gating strategies in flow cytometry. Some studies gated Tregs based only the expression of Foxp3^+^ CD25^+^ cells a population which contains non-Treg activated T cells. Recent studies using less ambiguous gating strategies reported that CD45RA^+^CD25^+^ naïve Treg and CD45RA^-^CD25^++^ activated Tregs in SLE patients are comparable to those in healthy individuals, although the frequency of CD45RA^-^CD25^+^ activated T cells showed linear relationship with SLEDAI (58). In addition, Foxp3^+^ T cells in the kidney and skin are comparable to those seen in tissues obtained from several control diseases. Considering that IL-2 production by T cells from SLE patients is impaired, it appears that this deficiency does not influence the numbers of Tregs in SLE. However, recent studies described that CD25 expression levels on the surface of Tregs were decreased in SLE patients (59). The reduction of CD25 expression in Tregs from patients with SLE correlated with the production of IL-2 by memory T cells indicating that deficiency of IL-2 in SLE patients reflects CD25 reduction in Tregs. Because IL-2 receptor-dependent activation of transcription factor STAT5 is essential for the suppressive function of Tregs, decreased expression of CD25 may affect the function of Tregs in SLE patients.

### IL-2 Therapy in Lupus-Prone Mice

The first report of IL-2 treatment for lupus-prone mice presented in 1990 prior to the discovery of Tregs (60). An IL-2-encoding vaccinia virus was used to deliver IL-2 *in vivo* in MRL*lpr* mice. Treated mice survived longer and had reduced lymphadenopathy and kidney pathology. As TCRαβ^+^CD4^-^CD8^-^ (double-negative, DN) T cells are the most likely culprit of lymphoadenopathy in MRL*lpr* mice, these DN T cells were significantly decreased after treatment with IL-2. Several methods for delivering IL-2 have been tried and confirmed these findings (61–63). Although DN T cells are also expanded in patients with SLE, their origin of is still unclear. DN T cells from lupus-prone mice and patients with SLE produce IL-17 (63, 64), indicating involvement of DN T cells in the pathogenesis of SLE. By IL-2 supplementation, Treg number is increased substantially in lymphoid and peripheral organs in NZB/NZW F1 mice and MRL/*lpr* mice and DN Tcells are significantly decreased in MRL/lpr mice (56, 63). However, Treg-specific expansion following the administration of IL-2/anti-IL-2 antibody complexes did not lead to the reduction of DN T cells (63), suggesting that an effect of IL-2 on non-Treg population might contribute to the inhibition of DN T cell expansion.

### IL-2 Therapy for Patients With SLE

Deficiency of IL-2 production in patients with SLE might contribute to detrimental perturbation in immune systems. Therefore, it is conceivable that low-dose IL-2 treatment can restore these pathogenic processes (65). Humrich and colleagues first reported a patient with SLE who achieved clinical improvement following treatment with low-dose IL-2. Specifically, 1.5 to 3 × 10^6^ IU IL-2 (aldesleukin) was injected subcutaneously on five consecutive days for four cycles with 9–16 days of separation. Skin eruption, myositis and arthritis were improved within 10 days and serum anti-dsDNA antibody titer was decreased after for cycles of treatment. CD4^+^CD25^+^Foxp3^+^CD127^lo^ Tregs were upregulated temporarily at around 40% among CD4^+^ T cells (11). Subsequently, they conducted a combined phase I/IIa clinical trial to address the safety, tolerability, efficacy, and immune response of low-dose IL-2 therapy in patients with active and refractory SLE (PRO-IMMUN, EudraCT-number: 2013-001599-40, Germany) (66). In this study, they demonstrated that Tregs from SLE patients showed decreased number of CD25^high^ population and that IL-2 production was deficient in SLE CD4^+^ T cells. After five patients were treated with daily subcutaneous injection of IL-2 at 1.5 × 10^6^ IU for 5 days, they confirmed that low-dose IL-2 therapy induced substantial increases of the numbers of Tregs without major side effects. As the primary endpoint (immune response rate) has been completed, phase II trial is now ongoing. The latest clinical trial of low-dose IL-2 in 38 SLE patients in China (NCT02084238) demonstrated that IL-2 treatment significantly decreased SLEDAI after 12 weeks (12). Subcutaneous 1 × 10^6^ IU of IL-2 was administered alternate-day for seven times at three cycles. More than 80% of patients achieved composite endpoint of SLE response index with 4-point drop in SLEDAI (SRI(4)), with increased Tregs, decreased T_H_17, Tfh, and DN T cells. Unfortunately, the study was not controlled and various observations including the rapid disappearance of DNA antibodies remain unexplained. Another clinical study involving the induction of Tregs by low-dose IL-2 in SLE and other autoimmune and inflammatory diseases (Charact-IL-2 and TRANSREG) is now in progress (67). Since all studies are non-controlled ones, controlled prospective study is necessary. Taken together, low-dose IL-2 treatment in SLE patients could alleviate clinical severity by altering the balance of T-cell subsets.

### Efficacy and Safety of Low-Dose IL-2 Therapy

Further analysis using mass cytometry of low-dose IL-2 treatment in cGVHD patients revealed that CD4^+^CD25^+^Foxp3^+^Helios^+^ Tregs and CD56^bright^CD16^-^ NK cells were selectively expanded (68). Helios^+^ Tregs were shown to be fully demethylated at the Treg-specific demethylated region and was recognized as a subset with enhanced suppressive potential (69). Ki67 expression was increased 1 week after starting IL-2 but declined to baseline after 12 weeks. It is notable that even 48 weeks after daily treatment with low-dose IL-2, phosphorylation of STAT5 and increased expression of Foxp3, CTLA-4, CD25, and Bcl-2 were sustained (68). A recent study reported that inflammation-experienced memory Tregs exert enhanced suppressive function which was lost over time to obviate general immunosuppression (70).

Long-term treatment with low-dose IL-2 has been tested in mice. Recombinant adeno-associated vector (rAAV) encoding IL-2 was injected intraperitoneally at various viral titers. This approach enabled sustained higher IL-2 concentrations for more than 20 weeks compared to controls and substantially prevented diabetes in NOD mice (71). Although mice injected with high viral titers (10^12^ rAAV IL-2) died within 2 weeks, mice injected with lower titer (10^9^–10^11^ rAAV-IL-2) lived normal life spans with unaffected vaccine-mediated antibody responses, infection-induced immune responses, and notably, not-enhanced tumor growth (71). Interestingly, low-dose recombinant IL-2 administration could protect mice from food allergy and the immune tolerance was sustained for more than 7 months after the last dose of IL-2 (72). These results indicate that Tregs can maintain their specific inhibitory function during long-term exposure to IL-2 and long thereafter.

### Manipulation of IL-2

Although low-dose IL-2 can substantially expand Tregs, frequent injection is required for the induction of significant increase because of its short half-life in human serum (5–7 min). To overcome this disadvantage, modified IL-2 such as polyethy-lene glycol-modified IL-2 (PEG-IL-2), which prolongs the half-life of IL-2, has been constructed. PEG-IL-2 has been developed in the 1990s and undergone phase I/II clinical trials in cancer patients (73) and was recently revisited and tried in mice with asthma (74). Bell et al. recently developed monovalent or bivalent IL-2-fused with non-FcR binding IgG1 molecules which had a prolonged half-life *in vivo* and caused prolonged activation and proliferation of Tregs after a single ultra-low dose (75). IL-2/anti-IL-2 complexes can also prolong the half-life of IL-2. In mice, IL-2/anti-IL-2 complexes have been well established: IL-2/JES6-1A12 specifically binds to CD25 and IL-2/S4B6 selectively binds to CD122. IL-2/JES6-1A12 and IL-2/S4B6 induce specific expansion of Tregs and cytotoxic lymphocytes, respectively. IL-2/JES6-1A12 administration was shown to expand efficiently both peripheral and tissue Tregs (43, 76). When human IL-2/anti-IL-2 complexes are fully developed, they will be useful for the specific expansion of target cells and will probably require less frequent injections (77). Biologic nanoparticles have attracted attention over the years for targeted therapy. For example, nanoscale liposomal polymeric gels (nanolipogels) are biologically compatible and slowly biodegradable agents. Fahmy and colleagues recently developed nanolipogels encapsulated recombinant IL-2 and TGFβ, and anti-CD4-labeled nanolipo-gels with IL-2 and TGFβ successfully expand Tregs *in vitro* and *in vivo* (78). Use of IL-2-nanoparticles tagged with an antibody recognizing specific tissues will result in bore specific delivery and lower toxicity.

## Targeting Metabolism to Increase Treg Stability

### Mechanistic Target of Rapamycin (mTOR)

Dynamic changes of cellular metabolism are necessary for efficient immune cell activation, growth, proliferation, and differentiation. In the quiescent state, T cells use mitochondrial tricarboxylic acid cycle to generate ATP and sustain homeostasis. When stimulated, cell metabolism shifts to anabolic pathways to produce building blocks needed to promote and sustain cell proliferation. Therefore, glycolytic metabolism is induced in activated T cells. The hosphatidylinositol-3-kinase (PI3K)–Akt–mTOR pathway plays a critical role in the regulation of glycolysis. Generally, resting Tregs utilize a distinct metabolic program based on mitochondrial oxidation of lipids (β-oxidation). When Tregs proliferate, glycolysis is also observed but their suppressive function is reduced. Conversely, Foxp3 inhibits the PI3K-Akt-mTOR pathway and glycolysis (79). mTOR consists of two multiprotein complexes (mTORC1 and mTORC2) and acts as a critical regulator of cell growth, metabolism, differentiation and survival. In mice with Treg-specific depletion of the regulatory-associated protein of mTOR, a component of mTORC1, Tregs lose their suppressive function resulting into severe autoimmunity (80). Inhibition of mTORC2 by mTORC1 has been shown to be important for Treg function and generation. On the other hand, mTORC1 inhibits *de novo* Treg differentiation and proliferation (81). Furthermore, uncontrolled activation of mTORC1 leads to the development of autoimmunity with deficiency of suppressive function of Tregs (82). Several studies with mice deficient in the mTOR regulatory systems showed functional impairment of Tregs that leads to systemic autoimmunity (82–85). Human Tregs have been reported to be expanded efficiently in the presence of the mTORC1 inhibitor rapamycin (86). In SLE, activated mTOR in T cells accounts for several abnormalities including the downregulation of CD3ζ, the expansion of T_H_17 and CD3^+^CD4^−^CD8^−^ DN T cells and the contraction of Tregs (87, 88). Administration of rapamycin has been reported to improve clinical outcomes in lupus-prone mice (89) and patients with SLE (90). Moreover, rapamycin can block the production of antiphospholipid antibody in lupus-prone mice (91) and enhance renal allograft survival of antiphospholipid syndrome patients (92). Therefore, rapamycin is a promising candidate for the treatment of patients with SLE because it normalizes various T cell functions including that of Tregs. Interestingly, inhibition of both mitochondrial electron transport by metformin and glucose metabolism by 2-deoxy-d-glucose (2DG) ameliorated disease in lupus-prone mice and cGVHD (93). Metformin can also inhibit mTORC1 by activating AMPK. Activated T cells, Tfh, and germinal center B cell and anti-dsDNA antibody titer were decreased, indicating that metabolic control can prevent aberrant activation of immune cells in autoimmunity (93).

### Calcium/Calmodulin-Dependent Kinase IV (CaMK4)

CaMK is a serine/threonine kinase family protein which becomes activated when intracellular calcium binds to calmodulin to generate the calcium/calmodulin complex. CaMK4 translocates in the nucleus and regulates the activation of several transcription factors including CREB and CREM (94). CaMK4 expression of SLE T cells is upregulated and CaMK4-deficient lupus mice show amelioration of autoimmunity with decreased T_H_17 and increased Treg cell numbers. Therefore, CaMK4 is involved in the pathogenesis of SLE by altering the balance between T_H_17 and Treg cells. Both T_H_17 and iTreg cells need TGFβ for their development. T cells expressing both Foxp3 and RORγt are generated intermediately and can differentiate to T_H_17 or iTreg dependent on the milieu. Therefore, T_H_17 and iTreg cells display plasticity which allows them to exchange phenotype. We recently reported that CaMK4 regulates T_H_17 cell differentiation by activating Akt–mTOR pathway as well as by enhancing CREMα-mediated IL-17 transcription (51). CaMK4 is preferentially expressed by T_H_17 cells and its deficiency mitigated the differentiation of naïve CD4^+^ T cells into T_H_17 cells. Because the mTOR–TORC1 pathway is essential for T_H_17 differentiation, CaMK4–Akt–mTOR axis might be critical for effective T_H_17 development. Moreover, administration of the CaMK4 inhibitor KN93 sufficiently expanded Tregs *in vivo* and alleviated disease in lupus-prone mice. Lastly, Otomo et al showed that anti-CD4-tagged nanoparticles loaded with KN93 selectively delivered the drug to CD4^+^ lymphocytes and mitigated disease in lupus-prone mice and in mice induced to develop experimental autoimmune encephalomyelitis (95).

## Treg Infusion Therapy

Several studies using animal models have demonstrated that adaptive transfer of natural or *ex vivo*-expanded Tregs can inhibit GVHD (96, 97) and solid organ transplant rejection (98). Treg infusion for human GVHD has been reported to be effective and currently a number of clinical trials involving the infusion of Tregs in patients receiving hematopoietic stem cell, kidney, and liver transplants are in progress (99). Since the number of Tregs which can be isolated from the peripheral blood or umbilical cord blood is limited, various strategies to expand Tregs *in vitro* have been considered including anti-CD3/CD28-coated beads in the presence of IL-2 and/or TGF-β and in the presence or absence of rapamycin (NCT02129881, NCT01624077). A recent study confirmed besides the efficacy, the safety, and feasibility of the injection of isolated or *ex vivo*-expanded Tregs in patients receiving transplant organs (100). Treg cell therapy is also ongoing in patients with type 1 diabetes (T1D) based on the evidence that deficiency of Tregs is important in the pathogenesis of the disease. A phase I trial (NCT01210664) designed to assess safety of adoptive Treg immunotherapy in patients with T1D has been completed. Specifically, T1D patients received *ex vivo*-expanded autologous CD4^+^CD127^low/-^CD25^+^ polyclonal Tregs (0.05 × 10^8^ to 26 × 10^8^ cells) using anti-CD3/CD28 beads plus IL-2. Twenty-five percent of transferred Tregs were detected even after one year without any adverse effect (101). A study involving the infusion of expanded autologous Tregs in a similar manner for the treatment of SLE has now been launched (NCT02428309). Since adaptive transfer of Treg in the setting of lupus-prone mice was reported effective in the suppression of glomerulonephritis and prolonging survival (102, 103), clinical efficacy is also expected from the trial in patients with SLE.

## Concluding Remarks and Future Perspectives

Sustained production of autoantibody and intermittent release of self-antigen can induce chronic activation of innate and adaptive immune responses in SLE. Chronic inflammatory conditions induce aberration of the immune system homeostasis. Further studies are necessary to elucidate the detailed mechanisms by which Tregs suppress autoimmune-associated tissue inflammation and regeneration.

**Figure 1:**
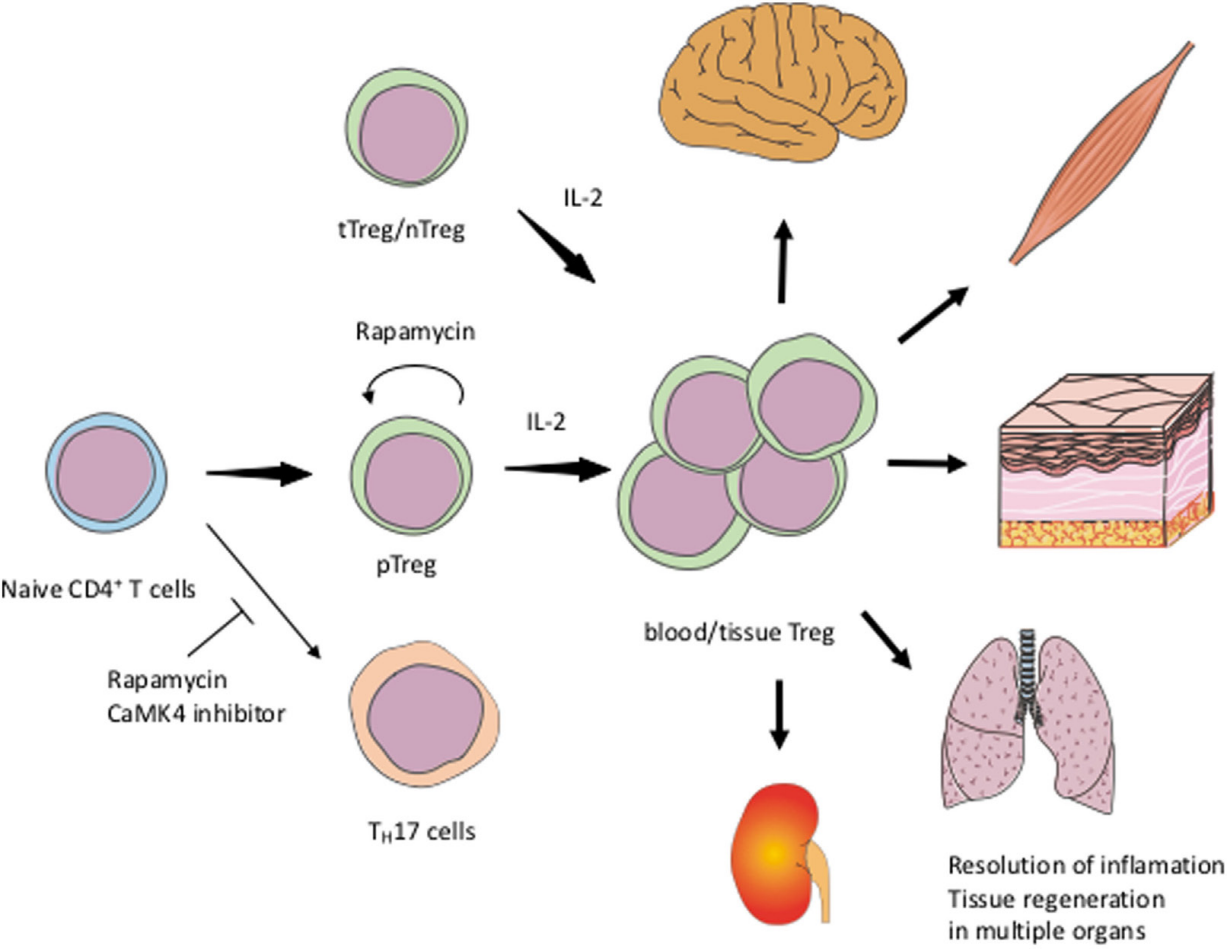
Schematic view of exploiting Tregs for the treatment of SLEIL-2 expands both tTreg/nTreg and pTreg including tissue Treg. Rapamycin and CaMK4 inhibitor facilitate the differentiation of Treg and inhibit T_H_17. Rapamycin can also stabilize the Treg function. tTreg, thymic regulatory T-cells; nTreg, naïve Treg; pTreg, peripheral Treg; T_H_17, IL-17-producing helper T-cells.

Low-dose IL-2 for the treatment of patients with SLE appears to be a promising, selective therapeutic strategy to expand Tregs numerically and functionally. Formulations of IL-2 to expand its half-life in the blood and to decrease the number of required injections are needed. Rapamycin and CaMK4 inhibitors would also be candidate drugs to enhance Treg function (Figure 1). IL-2/Sirolimus/Tacrolimus combination therapy was tried in patients undergoing hematopoietic stem cell transplantation with promising results (104). In mice, combination therapy of IL-2 and rapamycin effectively expanded Tregs and prevented acute rejection of skin grafts (105). Clinical trials of Treg-targeted treatments are currently in progress and it is expected to demonstrate that expansion or supplementation of Tregs can be added to the treatment choices physicians have in their disposal to treat patients with SLE.

## Author Contributions

MM and GT wrote and edited the review.

## Conflict of Interest Statement

The authors declare that the research was conducted in the absence of any commercial or financial relationships that could be construed as a potential conflict of interest.
